# Significant correlations between postoperative outcomes and various limb and component alignment strategies in medial unicompartmental knee arthroplasty: a systematic review

**DOI:** 10.1186/s40634-023-00655-3

**Published:** 2023-09-18

**Authors:** Manuel-Paul Sava, Alexandra Leica, Isabel Scala, Johannes Beckmann, Michael T. Hirschmann

**Affiliations:** 1 Department of Orthopaedic Surgery and Traumatology, Kantonsspital Baselland (Bruderholz, Liestal, Laufen), CH-4101 Bruderholz, Switzerland; 2 Department of Clinical Research, Regenerative Medicine & Biomechanics, Research Group Michael T. Hirschmann, University of Basel, CH-4001 Basel, Switzerland; 3 Faculty of Biomedical Sciences, University of Italian Switzerland (USI), CH-6900 Lugano, Switzerland; 4 Clinic for Orthopaedics and Traumatology, Krankenhaus Barmherzige Brüder München, 80639 Munich, Germany

**Keywords:** Medial UKA, Unicondylar knee arthroplasty, Partial knee replacement, Limb alignment, Component position, Coronal alignment, Rotational alignment, Systematic review

## Abstract

**Purpose:**

To investigate the correlation between postoperative limb/component alignments and clinical/functional outcomes following medial unicondylar knee arthroplasty (mUKA).

**Methods:**

Inclusion criteria included peer-reviewed English- or German-language publications assessing postoperative limb or implant alignment and clinical outcomes of mUKA. Methodological Index for Non-Randomized Studies (MINORS) was used to assess article quality.

**Results:**

A total of 2767 knees from 2604 patients were evaluated. Significant correlations were observed between postoperative limb/component alignments and clinical/functional outcomes after mUKA. Inferior outcomes were associated with lower placement and excessive valgus alignment of the tibia component (> 3°). A recommended external rotation of 4°-5° was identified for the tibia component, with specific cut-off values for the femoral and tibia components.

**Conclusions:**

Optimal outcomes in mUKA were associated with a varus coronal limb alignment. The tibia implant component performed well within a specific alignment range. An exact external rotation value was recommended for the tibia component, while internal rotation correlated negatively with the femoral component.

**Level of evidence:**

IV (level IV retrospective case series were included).

**Supplementary Information:**

The online version contains supplementary material available at 10.1186/s40634-023-00655-3.

## Background

Unicondylar knee arthroplasty (UKA) is a well-established treatment option for osteoarthritis and osteonecrosis, specifically targeting either the lateral or the medial compartments of the knee joint [5]. Long-term studies have shown that UKA offers advantages such as improved range of motion, preservation of knee kinematics, and faster recovery compared to total knee arthroplasty (TKA) [1]. However, registry data has indicated higher revision rates for UKA compared to TKA, primarily attributed to limb and component malalignment and the progression of arthritis to the contralateral side [2]. Consequently, the optimal alignment in UKA remains a topic of ongoing debate [19, 28].

Traditionally, patients undergoing medial UKA (mUKA) have a preoperative varus phenotype and overall varus limb alignment. However, recent research challenges this notion, suggesting a more nuanced understanding of knee phenotypes and alignments; Hirschmann et al. analyzed 308 non-osteoarthritic knees and identified 43 different knee phenotypes, with functional and anatomical alignment targets observed in varying proportions [12]. Furthermore, a wide range of femoral mechanical angle (FMA) values and tibial mechanical angle (TMA) values were observed [11]. Given the considerable variability in coronal alignment alone, the extent to which limb alignment influences mUKA outcomes and potential avenues for improving outcomes through alignment adjustments warrant investigation.

It is well known that undercorrection may contribute to increased polyethylene wear, while overcorrection may lead to osteoarthritis in the lateral knee compartment [9, 29]. Additionally, component position and alignment are believed to impact clinical and functional outcomes [3, 31]. Previous studies have cautioned against valgus alignment in the coronal plane and excessive posterior slope in the sagittal plane of the tibia component [4, 10].

The objectives of this systematic review are twofold: to identify significant correlations between different postoperative limb/component alignments and clinical/functional outcomes in mUKA, and to examine whether specific postoperative coronal limb or coronal/axial component alignments yield superior clinical and/or functional outcomes compared to alternative alignments. It is hypothesized that significant correlations between postoperative limb/component alignment and clinical/functional outcomes exist in mUKA. However, it is presumed that no single postoperative limb/component alignment strategy can unequivocally be proven superior in terms of clinical/functional outcomes.

## Materials and methods

A systematic literature search adhering to PRISMA guidelines [26] was conducted on PubMed, Embase, and Web of Science from their inception until September 2022 to identify potentially relevant articles for this review. Specific search terms such as “unicondylar knee replacement”, “unicondylar knee arthroplasty”, “unicondylar knee prosthesis”, “partial knee replacement”, “partial knee arthroplasty”, “partial knee prosthesis”, “unicompartmental knee replacement”, “unicompartmental knee arthroplasty”, “unicompartmental knee prosthesis”, “UKR”, “UKA”, “coronal alignment”, “clinical outcome”, “functional outcome” and “radiological outcome” were searched for in the title and abstract. Additional details regarding the search strategy can be found in Online Resource 1.

After removing duplicates and collecting all relevant articles, the studies were screened based on inclusion criteria using the title and abstract. Inclusion criteria encompassed English- or German-language publications in peer-reviewed journals that assessed the clinical and/or functional outcomes of medial unicondylar knee arthroplasty (mUKA) based on postoperative overall limb or component alignment. Studies unrelated to mUKA, pertaining to lateral unicondylar knee arthroplasty (lUKA), sagittal alignment, revision arthroplasty or failure rates were excluded. Only full-text articles with available numeric data (excluding graphical data) were considered.

Next, the selected articles were independently reviewed for eligibility through full-text analysis by two authors. The same authors then manually screened the reference lists for additional articles meeting the inclusion criteria but not covered by the original search terms. In case of uncertainty regarding inclusion a third author was consulted. The endpoints of the included studies comprised postoperative limb and component alignments, various clinical and functional scores, and patient-reported outcome measures (PROMs) such as the Knee Society Score (KSS), Oxford Knee Score (OKS), Knee Injury and Osteoarthritis Outcome Score (KOOS), Western Ontario and McMasters Universities Arthritis Index (WOMAC), and Forgotten Joint Score (FJS).

### Quality assessment

The methodological quality of the included studies was independently assessed by two authors using the Methodological Index for Non-Randomized Studies (MINORS) for non-randomized comparative and non-comparative clinical intervention studies [32]. The maximum ideal score was 16 for non-comparative studies and 24 for comparative studies. The level of evidence of the included studies was also reported. Varus values have been reported as positive angles and valgus ones as negative angles.

### Data extraction

Relevant information such as title, author, year of publication, study design, level of evidence, number of knees, follow-up period, patient demographics, clinical and functional outcome scores, and radiological outcomes were extracted from the selected publications by one author into a Microsoft Excel spreadsheet.

### Statistical analysis

Continuous variables were described using means and standard deviations or means and ranges, while categorical variables were reported as absolute and relative frequencies. A significance level of *p* < 0.05 was considered statistically significant for data interpretation.

## Results

**Fig. 1:**
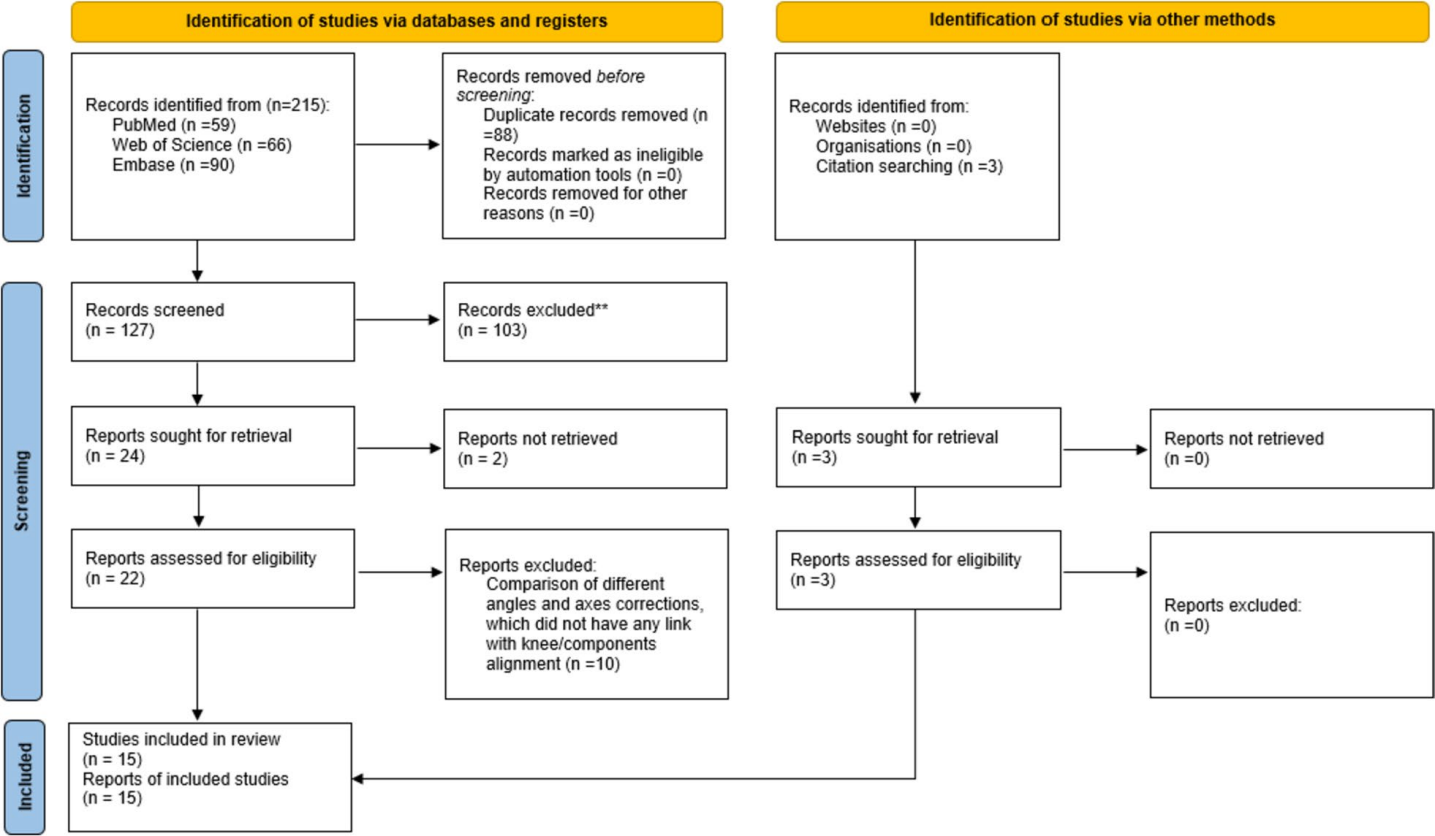
Flow-chart of the study selection *process* according to the PRISMA 2020 statement: an updated guideline for reporting systematic reviews [26]

The initial literature search yielded 215 publications, of which 12 met the inclusion criteria (Fig. 1). Three additional studies were identified through reference list screening. Table 1 presents the characteristics of the included studies.

**Table 1:** Overview of selected studies

Author (year)	Number of knees	Study type	Age mean (SD, range)	Gender male (%)	BMI mean (SD, range)	Follow-up time, mean (SD, range)	Evidence level	MINORS Score
Gill (2021) [6]	223 knees (223 patients)	Retrospective cohort	67.6 years (42–88)	52%	29.2 (4.0, 21–43)	2 years (nm)	III	12/16
Gulati (2009) [8]	211 knees (183 patients)	Prospective cohort	66.3 years (± 9.4, 36.1–85.7)	45%	28.1 (4.9, 14.9–44.2)	5.2 years (± 0.6, 4–7.9)	III	17/24
Gulati (2009) [7]	160 knees (160 patients)	Retrospective cohort	65.7 years (± 9.0)	45%	-	5 years (nm)	III	17/24
Inui (2020) [17]	52 knees (52 patients)	Prospective cohort	71.0 years (± 7.9)	29%	25.2 (3.7)	2.9 years (± 1.6)	III	15/16
Iriberri (2014) [18]	101 knees (88 patients)	Retrospective cohort	63.6 years (29–79)	40%	29.7 (20–40)	5.8 years (3–12.5)	III	17/24
Kamenaga (2018) [20]	45 knees (45 patients)	Case series	73.4 years (± 7.7)	29%	25.6 (3.4)	2 years (nm)	IV	14/16
Kamenaga (2018) [21]	50 knees (50 patients)	Retrospective cohort	72.9 years (± 6.7)	34%	25.9 (3.7)	2 years (nm)	III	14/16
Kennedy (2019) [22]	891 knees	Retrospective cohort	66.4 years (± 9.0)^a^	51%	81.5 (16.9)^ab^	10 years (5–17)	IV	19/24
Khow (2020) [23]	264 knees (213 patients)	Prospective cohort	61.0 years (± 7.6, 45–81)^a^	24%	27.2 (4.5)^a^	10 years (nm)	III	21/24
Kim (2012) [24]	246 knees (194 patients)	Retrospective cohort	61.5 years (± 7.1, 45–81)	2.8%	61.8 (7.4)^b^	7.4 years (± 1.3)	IV	17/24
Ng (2020) [25]	83 knees (67 patients)	Retrospective cohort	68.4 years (± 6.6, 55–87)	37%	27.2 (3.3, 22–38)	1.75 years (1.1–2.2)	III	18/24
Polat (2020) [27]	52 knees (49 patients)	Case series	60 years (49–80)	18%	34.6 (22–56.9)	4 years (2–6)	IV	11/16
Van der List (2016) [34]	143 knees (143 patients)	Retrospective cohort	65.4 years (± 9.4)	52%	27.2 (4.2)	2 years, (± 2.4; 2–5)	III	20/24
Yamagami (2020) [35]	142 knees (142 patients)	Retrospective cohort	72 years (54–88)	30%	25.1 (18.3–35.1)	2 years (nm)	III	22/24
Zuiderbaan (2016) [36]	104 knees (104 patients)	Case series	65 years (± 9.2, 45–84)	53%	33.2 (30.0–39.1)	2 years, (± 2.3, 2–3.7)	IV	12/16

*Abbreviations*: *BMI* body mass index (kilogram/meter^2^), *SD* standard deviation

^a^Values from multiple groups combined into one overall group

^b^Weight in kilogram

### Coronal limb alignment

When discussing WOMAC scores, it was observed that the 1–4° HKA group had better outcomes [36]. No significant differences were found in WOMAC and FJS scores between the -1–3° and 3–7° HKA groups [34]. Varus HKA alignments were however associated with lower OKS scores [7, 22], while the 0° to -2.5° tibiofemoral angle (TFA) group showed superior KSS scores [5]. Additionally, favorable KSS knee scores were found in the valgus HKA group [7, 24]. For detailed KSS and OKS data, please refer to Table 2.

**Table 2:** Reported KSS and OKS based on different HKA and FTA values

Author (year)	Measured angle postoperative	Groups	Number of knees (%)	KSS knee score^a^, mean ± SD	KSS function score^a^, mean ± SD	OKS^a^, mean ± SD
Gill (2021) [6]	TFA^b^	-10° to -7.5°	1 (0.5%)	90.0	100.0	
		-7.5° to -5°	4 (1.8%)	92.7 ± 10.0	94.3 ± 3.8	
		-5° to -2.5°	33 (14.8%)	97.5 ± 4.4*	96.3 ± 5.1	
		-2.5° to 0°	65 (29.1%)	96.0 ± 6.0*	96.2 ± 9.2	
		0° to 2.5°	71 (31.8%)	93.8 ± 8.9	94.9 ± 8.4	
		2.5° to 5°	37 (16.6%)	95.2 ± 4.8	96.9 ± 3.6	
		5° to 7.5°	9 (4.0%)	90.1 ± 13.7	93.8 ± 6.5	
		7.5° to 10°	3 (1.3%)	88.0 ± 7.2	93.0 ± 2.6	
Gulati (2009) [7]	HKA^b^	< 0°	13 (8.2%)	70 ± 13***(94 ± 8)^c^	92 ± 13	45.0 ± 4.0*
		0 to 4°	29 (18.4%)	77 ± 15*** (89 ± 14)^c^	86 ± 15.1	42.0 ± 5.0*
		5° to 10°	116 (73.4%)	94 ± 10*** (94 ± 10)^c^	83 ± 21	40.0 ± 9.0*
Kennedy (2019) [22]	HKA^b^	< 0°	67 (7.5%)	92.6 ± 11	81.6 ± 24	42.0 ± 7 (92%*)^d^
		0° to 4°	308 (34.6%)	90.7 ± 14	84.1 ± 17	41.1 ± 8 (85%*)^d^
		5° to 10°	508 (57.0%)	92.1 ± 12	84.6 ± 18	41.3 ± 8 (76%*)^d^
		> 10°	8 (0.9%)	-	-	
Kim (2012) [24]	TFA^b^	< 0°	11 (4.5%)	73.4 ± 7.6*** (90.6 ± 7.4)^c^	79.1 ± 10.4	
		1° to 3°	43 (17.8%)	80.9 ± 7.0*** (90.0 ± 6.6)^c^	82.6 ± 10.0	
		4° to 6°	101 (41.1%)	87.7 ± 8.5*** (88.5 ± 8.4)^c^	82.2 ± 12.5	
		7° to 9°	78 (31.7%)	88.3 ± 7.6*** (88.3 ± 7.6)^c^	80.3 ± 12.3	
		> 10°	13 (5.3%)	84.1 ± 14.1*** (85.8 ± 14.2)^c^	81.2 ± 17.1	

*Abbreviations*: *KSS* Knee Society Score, *OKS* Oxford Knee Score, *HKA* hip-knee-ankle angle, *TFA* tibiofemoral angle, *SD* standard deviation

^a^Scores at last follow-up

^b^Negative angles are varus and positive angles are valgus

^c^Score with deduction for other than neutral leg alignment and in brackets without deduction

^d^Percentages of good and excellent outcome in brackets

^*^ *p* < 0.05, *** *p* < 0.001

### Coronal implant alignment

The findings regarding the femoral component coronal angle (FCCA) were inconsistent, with varying optimal intervals for KSS and OKS scores reported across studies (Table 3). However, for the tibia component coronal angle (TCCA), most authors identified the -2.5° to 5° range as associated with the best clinical and functional outcomes (Table 4). In the study by Kamenaga et al. [20], a negative correlation was found between different tibia component angles and heights with OKS and OKS recovery, indicating worse outcomes for lower-placed tibia components (Table 5).

**Table 3:** Reported KSS and OKS based on different FCCA values

**Author (year)**	**Measured angle**	**Groups**	**Number of knees**	**KSS knee score**^a^**, mean ± SD**	**KSS function score**^a^**, mean ± SD**	**OKS**^a^**, mean ± SD**
Gill (2021) [6]	FCCA^b^	< -7.5°	4 (1.8%)	99.0 ± 4.7	96.3 ± 5.4	
		-7.5° to -5°	8 (3.6%)	94.0 ± 5.2	96.6 ± 6.6	
		-5° to -2.5°	28 (12.6%)	94.2 ± 9.0	95.4 ± 7.5	
		-2.5° to 0°	63 (28.3%)	94.2 ± 9.2	96.2 ± 6.9	
		0° to 2.5°	69 (30.9%)	95.6 ± 6.9	95.7 ± 8.9	
		2.5° to 5°	37 (16.6%)	96.8 ± 4.5	97.4 ± 3.5	
		5° to 7.5°	10 (4.5%)	94.2 ± 7.8	90.7 ± 12.6	
		> 7.5°	4 (1.8%)	97.5 ± 3.7	94.5 ± 2.9	
Gulati (2009) [7]	FCCA^b^	-10° to -7.5°	3 (1.4%)			37.3 ± 15.1
		-7.5° to -5°	8 (3.8%)			38.6 ± 9.4
		-5° to -2.5°	25 (11.8%)			42.4 ± 6.3
		-2.5° to 0°	38 (18.0%)			39.9 ± 7.5
		0° to 2.5°	65 (30.8%)			40.2 ± 8.2
		2.5° to 5°	31 (14.7%)			40.6 ± 8.8
		5° to 7.5°	27 (12.8%)			38.5 ± 9.6
		7.5° to 10°	11 (5.2%)			43.7 ± 3.6
		10° to 12.5	3 (1.4%)			38.3 ± 16.7
Khow (2020) [23]	FCCA^b^	≤ 3° (mean 1.6°)	106 (40.2%)	85.4 ± 12.6	75.0 ± 18.8	17.8 ± 3.4^c^
		> 3° (mean 6.6°)	158 (59.8%)	82.4 ± 18.9	74.2 ± 18.8	19.6 ± 7.8^c^*

*Abbreviations*: *KSS* Knee Society Score, *OKS* Oxford Knee Score, *FCCA* femoral component coronal angle, *SD* standard deviation, *ns* no statistically significant difference

^a^Scores at last follow-up

^b^Negative angles are varus and positive angles are valgus

^c^Higher scores indicate worse outcome

^*^p < 0.05

**Table 4:** Reported KSS and OKS based on different TCCA and TPA intervals

**Author (year)**	**Measured angle**	**Groups**	**Number of knees**	**KSS knee score**^a^**, mean ± SD**	**KSS function score**^a^**, mean ± SD**	**OKS**^a^**, mean ± SD**
Gill (2021) [6]	TCCA^b^	-12.5° to -10°	3 (1.3%)	96.7 ± 21	98.0 ± 3.5	
		-10° to -7.5°	10 (4.5%)	92.7 ± 10.1	92.0 ± 8.9	
		-7.5° to -5°	22 (9.9%)	94.5 ± 9.5	94.3 ± 13.3	
		-5° to -2.5°	55 (24.6%)	94.8 ± 8.9	95.5 ± 7.0	
		-2.5° to 0°	77 (34.5%)	95.6 ± 6.0	96.6 ± 6.4	
		0° to 2.5°	48 (21.5%)	95.3 ± 7.1	96.2 ± 6.1	
		2.5° to 5°	7 (3.1%)	96.4 ± 4.8	97.9 ± 2.3	
		5° to 7.5°	1 (0.4%)	80.0	90.0	
Gulati (2009) [7]	TCCA^b^	-7.5° to -5°	18 (8.5%)			38.9 ± 7.6
		-5° to -2.5°	80 (37.9%)			39.5 ± 9.1
		-2.5° to 0°	76 (36.0%)			41.7 ± 6.9
		0° to 2.5°	32 (15.2%)			39.4 ± 9.3
		2.5° to 5°	5 (2.4%)			41.8 ± 4.6
						ns
Polat (2020) [27]	TPA^c^	> 90°	2 (3.8%)	54.0 ± 32.5	45.0 ± 63.6	21.0 ± 12.7
		90°	34 (65.4%)	88.5 ± 17.0**	84.4 ± 19.3*	38.8 ± 9.6*
		85° to 89°	11 (21.2%)	94.7 ± 6.6**	92.3 ± 11.7*	42.9 ± 3.3*
		< 85°	5 (9.6%)	59.4 ± 25.2	58.0 ± 37.7	26.4 ± 12.4

*Abbreviations*: *KSS* Knee Society Score, *OKS* Oxford Knee Score, *TCCA* tibia component coronal angle, *TPA* tibia plateau angle, *SD* standard deviation, *ns* no statistically significant difference

^a^Scores at last follow-up

^b^Negative angles are varus and positive angles are valgus

^c^Angle < 90° is varus and > 90° valgus

^*^p < 0.05, **p < 0.01

**Table 5:** Correlations between KSS, OKS and coronal component alignment intervals

**Author (year)**	**Measured angles**	**Scores**^a^	**Significant Correlations**^b^
Kamenaga (2018) [20]	AKI^c^ TOL^c^ TCH-I^d^ TCH-L^d^	OKS OKS recovery	AKI and OKS: -0.34* AKI and OKS recovery: -0.4** TOL and OKS: -0.4* TOL and OKS recovery: -0.53** TCH-I: and OKS recovery: -0.46* TCH-L and OKS: -0.41* TCH-L and OKS recovery: -0.51**
Khow (2020) [23]	FCCA^c^	KSS KS KSS FS OKS 2 years^e^ OKS 10 years^e^	FCCA and OKS 2 years: 0.266** FCCA and OKS 10 years: 0.296*

*Abbreviations*: *KSS* Knee Society Score, *OKS* Oxford Knee Score, *AKI* ankle-knee-implant angle, *TOL* tibia component obliquity relative to the lateral compartment, *TCH-I* tibia component height relative to the intercondylar eminence, *TCH-L* tibia component height relative to the lateral joint, *FCCA* femoral component coronal angle, *SD* standard deviation, ns: no statistically significant difference

^a^Scores at last follow-up

^b^Linear regression analysis

^c^Negative angles are varus and positive angles are valgus

^d^Higher values indicates a lower placement of the tibia component

^e^Higher scores indicate worse outcome

^*^p < 0.05, **p < 0.01

### Axial implant alignment

When examining the tibia component, higher values of external rotation were linked to lower KSS and OKS scores (Table 6). Ng et al. [25] established significant cut-off values of 8–9° for the femoral component and 10–12° for the tibia component, indicating an impact on KSS and OKS scores. Studies conducted by Kamenaga et al. [21], Ng et al. [25], and Inui et al. [17] investigated the relationship between axial component angles and patient-reported outcomes, revealing negative associations between tibia component external/internal rotations and outcome scores (Table 7).

**Table 6:** Reported KSS and OKS based on different postoperative axial alignment of the components

**Author (year)**	**Measured angles**	**Mean ± SD (range)**^b^	**KSS knee score**^a^**, mean ± SD (range)**	**KSS function score**^a^**, mean ± SD**	**OKS**^a^**, mean ± SD**	**OKS recovery**^a^
Inui (2020) [17]	RFC RTC TIR extension TIR flexion 90°	-2.0° ± 3.8 (-4.0 to 9.6) 0.0° ± 4.1 (-12.5 to 8.6) -0.3° ± 6.4 5.4° ± 6.4	86.9 ± 9.4	81.8 ± 16.4		
Iriberri (2014) [18]	Tibia ER	11.9° (-1 to 32)	79 (28–100)	79 (5–100)		
Kamenaga (2018) [21]	Alpha Beta	4.0° ± 4.6 (-6.4 to 12.7) 2.43° ± 4.15 (-5.6 to 9.8)		80.4 ± 15.3	37.2 ± 7.9	10.2 ± 8.0
Ng (2020) [25]	Femoral ER Tibia ER TIR extension	4.8° ± 3.6 (0 to 25) 7.5° ± 5.5 (-5 to 20.1) 2.7° ± 6.8 (-13.8 to 17.8)	92.33 ± 7.39	73.27 ± 15.41	39.71 ± 3.33	

*Abbreviations*: *KSS* Knee Society Score, *OKS* Oxford Knee Score, *RFC* rotational femoral component angle, *RTC* rotational tibia component angle, *TIR* tibia component internal rotation angle relative to the femoral component, *Alpha* angle between component and Akagi’s line, *Beta* angle between component and line perpendicular to surgical epicondyles axis (SEA), *IR* internal rotation, *ER* external rotation, *SD* standard deviation

^a^Scores at last follow-up

^b^Negative values are IR and positive values are ER

**Table 7:** Significant Correlations between several axial component alignment angles, KSS, and OKS

**Author (year)**	**Measured angles**	**Scores**^a^	**Significant Correlations**^b^
Inui (2020) [17]	RFC RTC TIR extension TIR flexion 90°	KSS KS KSS FS KOOS	TIR flexion 90° and KSS FS: -0.32* TIR flexion 90° and KOOS pain: -0.34*
Kamenaga (2018) [21]	Alpha Beta	KSS FS OKS OKS recovery	Alpha and KSS FS: -0.52* Alpha and OKS: -0.54* Alpha and OKS recovery: -0.55* Beta and KSS FS: -0.34* Beta and OKS: -0.49* Beta and OKS recovery: -0.52*
Ng (2020) [25]	Femoral ER Tibia ER TIR extension	KSS KS KSS FS OKS	Tibia ER and KSS KS: -0.1* Tibia ER and KSS FS: -0.13** Tibia ER and OKS: -0.16** TIR and OKS: -0.06*

*Abbreviations*: *KSS* Knee Society Score, *OKS* Oxford Knee Score, *RFC* rotational femoral component angle, *RTC* rotational tibia component angle, *TIR* tibia component internal rotation angle relative to the femoral component, *Alpha* angle between component and Akagi’s line, *Beta* angle between component and line perpendicular to surgical epicondylar axis (SEA), *IR* internal rotation, *ER* external rotation, *SD* standard deviation, ns: no statistically significant difference

^a^Scores at last follow-up

^b^Linear regression analysis

^*^p < 0.05, **p < 0.01

## Discussion

The main finding of this review is the presence of significant correlations between different limb/component alignments and clinical/functional outcomes following mUKA. Specifically, the 1º-4º HKA alignment was reported as an optimal range for the coronal alignment of the knee, resulting in superior functional and clinical outcomes compared to other analyzed intervals. However, findings regarding the coronal alignment of the femoral component are somewhat heterogeneous and ambiguous, with different values presented by multiple authors as yielding the best outcomes. On the other hand, when discussing the coronal alignment of the tibia component, the analyzed studies predominantly suggest the -2.5º (valgus)—5º (varus) interval as generating the best clinical and functional outcomes. Furthermore, worse outcomes have been observed for patients with a tibia component positioned lower than the intercondylar eminence and the lateral joint, and/or in excessive valgus alignment relative to the lower limb axis. In terms of the axial alignment of the implant, a clear recommended interval of external rotation for the tibia component has been identified as 4º-5º. Cut-off values for external rotation of the tibia and femoral components have also been determined, with 10º-12º for the femoral component and 8º-9º for the tibia component. Additionally, a negative correlation has been observed between internal rotation of the tibia component and the femoral component. In conclusion, the study has achieved its aims and partially confirmed the hypothesis.

One interesting finding is the debatable reliability of the Knee Society Score (KSS) in calculating clinical outcomes, particularly the clinical/objective KSS. Several included studies question the deduction of KSS scores for varus or valgus limb alignments that fall outside the recommended intervals, such as 5º-10º valgus FTA for the 1989 KSS or 2º-10º valgus for the 2011 KSS [13, 14, 16, 30]. Authors such as Gulati et al. [7], Kim et al. [24], and Kennedy et al. [22] have reported poorer clinical/objective KSS scores associated with increasing valgus alignments solely because points were deducted for alignment values outside the recommended interval. Furthermore, patient satisfaction, pain (VAS), and functional scores (WOMAC, OKS) did not align with the clinical/objective KSS values (1989/2011), as some patients with inferior clinical/objective KSS scores exhibited superior WOMAC, OKS, and VAS scores. The use of different KSS scores by the authors of the included studies contributes significantly to the contradictory nature of some of the results.

This systematic review is the first to analyze both limb and implant component alignments following mUKA from the perspective of clinical and functional outcomes. While Riviere et al. conducted a systematic review on limb alignment in mUKA [28]; their focus on kinematic alignment led them to exclude any alignment strategy different from the kinematic approach. Additionally, they did not describe specific limits for the kinematic mUKA alignment technique. Furthermore, they are the only authors who use the term "kinematic alignment" in the context of medial UKA, making a direct comparison between their results and ours impossible.

However, this study has several limitations. One drawback is the lack of differentiation between fixed and mobile bearing medial UKA. The risks associated with overstuffing the medial compartment in mobile bearing UKA, as highlighted by Smith et al. [33], were not analyzed. Conversely, mobile bearing mUKA carries the risk of overcorrection, where a neutral to minor valgus alignment may lead to the initiation or progression of arthritic changes in the lateral compartment [15]. The loss of tension in the lateral collateral ligament or medial collateral ligament, which can result in bearing dislocation, especially in mobile bearing UKA, was also not discussed. Additionally, while there has been an increase in the number of papers on mUKA, long-term follow-up data in the literature are still limited. Moreover, the quality of the included studies is not very high, as no randomized controlled trials (RCTs) were available, and most studies were retrospective cohorts or case series. The retrospective nature of the majority of the studies included may have introduced patient selection bias, leading to potentially misleading results. The results also exhibit a significant degree of heterogeneity.

Despite these limitations, the reported results should provide guidance to orthopedic surgeons and improve the understanding of mUKA as a valid option for reducing knee pain and restoring functionality in patients with isolated medial osteoarthritis. However, future studies with higher levels of evidence and larger cohorts are needed. An international conversation should also be initiated regarding the criteria used in the KSS to award or deduct points for knee alignment. Additionally, given the existence of two KSS scores (1989 and 2011) in circulation, it is recommended that orthopedic surgeons reach a consensus and recommend the use of only one KSS while discontinuing the other. This will help prevent the dissemination of heterogeneous and contradictory results in the scientific community.

## Conclusion

Optimal outcomes in mUKA were associated with a varus coronal limb alignment. The tibia implant component performed well within a specific alignment range. An exact external rotation value was recommended for the tibia component, while internal rotation correlated negatively with the femoral component.

### Supplementary Information

**Additional file 1. **

## Data Availability

The dataset supporting the conclusions of this article is included within the article and its additional files.

## Abbreviations

FJA: Femoral mechanical angle
FJS: Forgotten Joint Score
FCCA: Femoral component coronal angle
FMA: Femoral mechanical angle
HKA: Hip-knee-ankle
KSS: Knee Society Score
KOOS: Knee Injury and Osteoarthritis Outcome Score
lUKA: Lateral unicondylar knee arthroplasty
MINORS: Methodological Index for Non-Randomized Studies
mUKA: Medial unicondylar knee arthroplasty
OKS: Oxford Knee Score
PRISMA: Preferred Reporting Items for Systematic Reviews and Meta-Analyses
PROMs: Patient-reported outcome measures
TFA: Tibiofemoral angle
TCCA: Tibia component coronal angle
TKA: Total knee arthroplasty
UKA: Unicondylar knee arthroplasty
UKR: Unicompartmental knee replacement
VAS: Visual Analog Scale
WOMAC: Western Ontario and McMaster Universities Arthritis Index
