# Supplementary material for: Significant correlations between postoperative outcomes and various limb and component alignment strategies in medial unicompartmental knee arthroplasty: a systematic review
Source: J Exp Orthop. 2023 Sep 18;10:93. doi: 10.1186/s40634-023-00655-3 (PMC10505601; doi:10.1186/s40634-023-00655-3)
Supplement: Supplementary file 1 — Additional file 1. [file 40634_2023_655_MOESM1_ESM.docx]

Search conducted on 22^nd^ of September 2022

Embase

('unicompartmental knee arthroplasty' OR 'unicompartmental knee prosthesis' OR 'unicompartmental knee replacement' OR 'unicondylar knee replacement' OR 'unicondylar knee arthroplasty' OR 'unicondylar knee prosthesis' OR 'partial knee arthroplasty' OR 'UKA' OR 'UKR') AND ('alignment') AND ('clinical outcome' OR 'functional outcome' OR 'radiological outcome')

Pubmed and WOS

("unicondylar knee replacement" OR "unicondylar knee arthroplasty" OR "unicondylar knee prosthesis" OR "partial knee replacement" OR "partial knee arthroplasty" OR "partial knee prosthesis" OR "unicompartmental knee replacement" OR "unicompartmental knee arthroplasty" OR "unicompartmental knee prosthesis" OR "UKR" OR "UKA") AND ("alignment") AND ("clinical outcome" OR "functional outcome" OR "radiological outcome")
